# Association between Sensitivity to Thyroid Hormone Indices and Bone Mineral Density in US Males

**DOI:** 10.1155/2022/2205616

**Published:** 2022-10-26

**Authors:** Shuai Chen, Wucui Huang, Guowei Zhou, Xiaohe Sun, Jie Jin, Zhiwei Li

**Affiliations:** ^1^Department of Orthopaedics, The Second Affiliated Hospital of Nanjing University of Chinese Medicine, Nanjing, China; ^2^Department of Respiratory and Critical Care Medicine, Zhongda Hospital Affiliated to Southeast University, Nanjing, China; ^3^Department of General Surgery, Jiangsu Province Hospital of Chinese Medicine, Affiliated Hospital of Nanjing University of Chinese Medicine, Nanjing, China; ^4^Department of Oncology, Jiangsu Province Hospital of Chinese Medicine, Affiliated Hospital of Nanjing University of Chinese Medicine, Nanjing, China

## Abstract

**Objectives:**

Thyroid hormone is acknowledged as a pivotal factor in skeletal development and adult bone maintenance. However, available data about the relationship between sensitivity to thyroid hormone and bone mineral density (BMD) remain limited and conflicting. The purpose of the study was to explore the complex relationship between sensitivity to thyroid hormone indices and BMD using cross-sectional analysis.

**Methods:**

An overall sample of 3,107 males from the National Health and Nutrition Examination Survey (NHANES) was studied in the study. The thyroid hormone sensitivity indices included free triiodothyronine/tree thyroxine (FT3/FT4), thyroid-stimulating hormone index (TSHI), thyrotroph thyroxine resistance index (TT4RI), and thyroid feedback quantile-based index (TFQI). Given the complex study design and sample weights, the correlation between sensitivity to thyroid hormone indices and BMD was evaluated through multivariate linear regression models, and extra subgroup analyses were performed to examine the robustness of the results.

**Results:**

Among the 3,107 participants, we demonstrated that FT3/FT4 was negatively correlated with lumbar BMD (*β* = −0.0.35, 95% CI: −0.084–0.013, *P* < 0.05). In the terms of central sensitivity to thyroid hormone, TFQI showed a significant negative relationship with the BMD of the lumbar (*β* = −0.018, 95% CI: −0.033 to −0.003, *P* < 0.05), total femur (*β* = −0.020, 95% CI: −0.035 to −0.006, *P* < 0.01), and femur neck (*β* = −0.018, 95% CI: −0.031 to −0.005, *P* < 0.01). In the subgroup analyses stratified by body mass index (BMI), the significant negative correlation between TFQI and lumbar BMD remained in the male participants with BMI between 18.5 and 24.9 kg/m^2^.

**Conclusions:**

Decreased indices of sensitivity to thyroid hormones are strongly associated with increased lumbar BMD, suggesting that the dysfunction of peripheral and central response to thyroid hormone might contribute to bone loss. In addition, FT3/FT4 and TFQI were considered to be the preferable indicators to guide the prevention and clinical treatment of osteoporosis.

## 1. Introduction

Osteoporosis is a metabolic bone disease characterized by the degeneration of bone tissue microstructure and decreased bone mineral density. The early clinical manifestations of osteoporosis are subtle, but many patients often have clinical manifestations such as spinal deformity, bone pain, and fractures in the later stage of osteoporosis [1, 2]. With the aging population, osteoporosis has already become an increasingly significant public health problem [3]. According to data released by the Centers for Disease Control and Prevention (CDC), osteoporosis-related medical expenditures are on the rise globally and the costs of osteoporotic fractures are expected to more than double by 2050 if there are no effective interventions for the condition in question [4, 5].

Osteoporosis includes primary osteoporosis and secondary osteoporosis, closely related to metabolic abnormalities, gene polymorphisms, and microcirculation disorders [6, 7]. As an endocrine disorder, thyroid diseases are the leading causes of secondary osteoporosis [8]. Viapiana et al. found that hyperthyroidism causes a high bone turnover by accelerating both bone formation and resorption phases [9]. As soon as the balance between bone remodeling and bone resorption was disrupted, there would be reduced bone mass and even osteoporosis [10]. In addition, several studies reported that thyroid hormone deficiency can slow the stimulation of osteoblasts and osteoclasts, thereby severely impairing bone gain during growth and development [11].

Thyroid function is evaluated clinically by measuring serum-free triiodothyronine (FT3), free thyroxine (FT4), and thyroid-stimulating hormone (TSH) levels. However, the complex relationship among these three indicators can be measured with sensitivity to thyroid hormone indices, which provide a comprehensive explanation of thyroid status [12]. Currently, several studies have reported that TT4RI, TSHI, and TFQI can be used as quantitative indicators of pituitary thyrotropin function in the central pituitary [13, 14]. In peripheral tissues, FT4 is converted to FT3 by deiodinase. Therefore, FT3/FT4 is not only an indicator of deiodination activity but can also indirectly reflect the peripheral sensitivity to thyroid hormones [15, 16]. Laclaustra proposed TFQI and simultaneously used TSHI and TT4RI indices to explore the interaction between impaired sensitivity to thyroid hormones and metabolic diseases [17]. However, it is unclear whether sensitivity to thyroid hormone is related to bone metabolism. Therefore, we examined the independent correlation between central and peripheral thyroid hormone sensitivity and bone mineral density (BMD) using NHANES (2007–2010) data.

## 2. Materials and Methods

### 2.1. Study Population

The NHANES is an ongoing cross-sectional population-based survey conducted by the National Center for Health Statistics (NCHS) at the Centers for Disease Control (CDC) to assess the health and nutritional status of individuals in the US. Informed consents for data collection were obtained from all participants.

**Figure 1:**
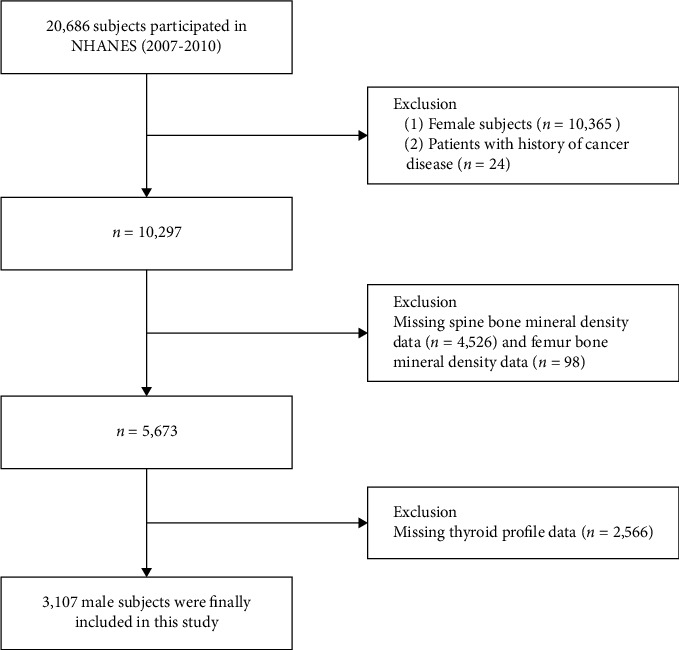
Study flowchart.

Data collected included demographic, laboratory, and examination information from the NHANES database from 2007 to 2010. Study participants who had completed demographics, thyroid parameters, and BMD measurements (*n* = 20,686) were included. Exclusion criteria included females and patients with a history of cancer diseases (*n* = 10,389), missing BMD data (*n* = 4,624), and missing thyroid profile data (*n* = 2,566). Ultimately, a total of 3,107 male subjects were included in the analysis (Figure 1).

### 2.2. Assessment of BMD

Dual-energy X-ray absorptiometry (DXA) is the standard method of measuring bone density, due in part to its speed, ease of use, and low radiation exposure [18, 19]. Beginning in 2005, DXA scans of the lumbar spine have been administered in the NHANES mobile examination center (MEC). As the exposure variables of this study, total femur, femoral neck, and lumbar spine BMD were estimated by DXA with a Hologic QDR-4500A fan-beam densitometer (Hologic, Inc., Bedford, Massachusetts).

### 2.3. Assessment of Thyroid Function

The TSH level was detected by a third-generation, two-site immunoenzymatic (“Sandwich”) assay (reference range, 0.34–5.6 *μ*IU/ml) [20]. The FT3 assay was a competitive binding immunoenzymatic assay (reference range, 2.5–3.9 pg/ml) [21], and the FT4 assay was a two-step enzyme immunoassay (reference range, 0.6–1.6 ng/dl) [22]. Detailed specimen collection and processing instructions are available in the NHANES Laboratory/Medical Technologists Procedures Manual (LPM).

### 2.4. Definition of Sensitivity to Thyroid Hormone Indices

Sensitivity to thyroid hormone indices include FT3/FT4, TT4RI, TSHI, and TFQI.

1. FT3/FT4 = FT3 (pmol/L)/FT4 (pmol/L) [23]
2. TSHI = ln TSH (mIU/L) + 0.1345^*∗*^FT4 (pmol/L) [14]
3. TT4RI = FT4 (pmol/L)^*∗*^TSH (mIU/L) [24]
4. TFQI = cdfFT4 − (1−cdfTSH)

The TFQI was calculated by the empirical cumulative distribution function to evaluate the central sensitivity to thyroid hormone; the TFQI value ranges from −1 to 1 [17]. According to the previous studies, 0 indicated that the sensitivity of the HPT axis to FT4 was normal, and the negative value suggested that the HPT axis was more sensitive to changes in FT4, while the positive value indicated the insensitive response [17].

### 2.5. Covariates

Demographic information included age (mean age, 39.48 years; age range, 12–80 years), race (non-Hispanic white, non-Hispanic black, Mexican American, and other races, including other Hispanic and multiracial), and education level (less than 9th grade, 9–11th grade, high school grad, some college, college graduate or above, and missing). Laboratory data included glycosylated hemoglobin A1c (HbA1c, %), total calcium (mg/dL), phosphorus (mg/dL), cholesterol (mg/dL), triglycerides (mg/dL), albumin (g/dL), blood urea nitrogen (mg/dL), creatinine (mg/dL), and uric acid (mg/dL). Finally, questionnaire data extracted included alcohol consumption, smoking behavior, BMI, hypertension, and diabetes. Smoking behavior was defined as significant smoking behavior and never or nonsignificant smoking. Significant smoking behavior was defined by 100 cigarettes in life either current or in the past and never or nonsignificant smoking as participants who smoke less than 100 cigarettes [25]. Alcohol consumption was classified as significant alcohol intake and nonsignificant alcohol intake. Significant alcohol intake is defined as 12 units per week and nonsignificant alcohol intake as participants who drink less than 12 units per week [26]. Based on the 2018 Physical Activity Guidelines for Americans, participants without any physical activity, with physical activity of more than 0 minutes per week (min/wk) but less than 150 min/wk, and with physical activity of 150 min/wk or more during the previous week were classified as light, moderate, and high, respectively [27, 28]. The BMI classification is defined as below 18.5: underweight, 18.5–24.9: healthy weight, 25.0–29.9: overweight, and 30.0 and above: obesity [29]. The diagnostic criteria of “diabetes” and “hypertension” were supplemented in the covariable. “Hypertension” was defined as having an SBP ≥ 140 or/and DBP ≥90 mmHg or a history of hypertension [30]. Diabetes status was confirmed when the participants had a positive history of diabetes and/or elevated glycosylated hemoglobin (HbA1c) > 6.5% [31].

### 2.6. Statistical Analyses

Data that conformed to normal and skewed distributions were expressed as the mean standard deviation for continuous variables, while categorical variables were expressed as the number and percentage of subjects [32]. Subjects were divided into quartiles based on BMD levels (range from 0.423 to 1.901 g/cm^2^), and univariate analysis and chi-square tests were used to examine correlations between research variables and BMD. We explored the association between sensitivity to thyroid hormone indices and BMD in three different models using weighted multivariate linear regression models. Model 1 had no variables adjusted. Model 2 was adjusted for age, race, and education. Model 3 was further adjusted for age, race, education, physical activity, smoking behavior, alcohol behavior, BMI, hypertension, diabetes, HbA1c, total calcium, phosphorus, cholesterol, triglycerides, albumin, blood urea nitrogen, creatinine, and uric acid. Furthermore, we conducted subgroup analyses to examine the robustness of the association between TFQI and BMD. Finally, we used smooth curve fittings and generalized additive models to address the nonlinear relationship. A *P* value of <0.05 was considered statistically significant. All analyses were performed with R software and EmpowerStats.

## 3. Results

### 3.1. Baseline Characteristics of the Participants

Sociodemographic and clinical characteristics of 3,107 male participants based on lumbar BMD quartiles are presented in Table 1. There were significant differences in age, race, education levels, smoking behavior, alcohol consumption, and hypertension across all quartiles of lumbar BMD (*P* < 0.001). Participants with higher lumbar BMD levels also had significantly higher levels of BMI, HbA1c, blood urea nitrogen, triglycerides, creatinine, and uric acid. While, their total calcium, albumin, and phosphorus levels were significantly lower (*P* < 0.001). In addition, FT3, FT3/FT4, and TFQI tended to be lower in patients with higher lumbar BMD (*P* < 0.001).

### 3.2. Correlation between Sensitivity to Thyroid Hormone Indices and BMD

The results of the weighted multivariate linear regression analyses are reported in Table 2. After adjusting confounding factors, we found that FT3/FT4 and TFQI levels were negatively correlated with lumbar BMD in model 3 (*β* = −0.0.35, 95% CI: −0.084 to −0.013, *P* < 0.05) and (*β* = −0.018, 95% CI: −0.033 to −0.003, *P* < 0.05). And the association between TFQI and BMD was also significant at the total femur (*β* = −0.020, 95% CI: −0.035 to −0.006, *P* < 0.01) and femur neck (*β* = −0.018, 95% CI: −0.031 to −0.005, *P* < 0.01). Furthermore, after transforming TFQI to a categorical variable (quartiles), as compared with the Q1 group, the TFQI levels of the Q2 and Q4 groups were still negatively correlated with lumbar BMD. The male participants in the Q4 group had a 0.012 g/cm^2^ lower lumbar BMD than those in the Q1 group. And the trend remained significant among different TFQI quartile groups (*P* for trend < 0.01) (Table 3).

### 3.3. Associations between FT3/FT4, TFQI, and Lumbar BMD

**Figure 2:**
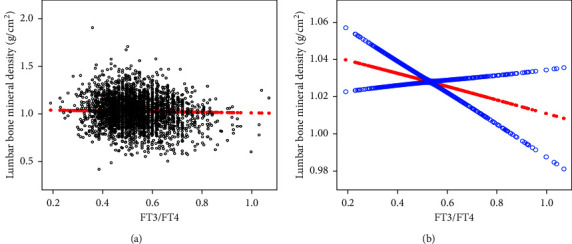
The association between FT3/FT4 and lumbar BMD. (a) Each black point represents a sample. (b) Solid red line represents the smooth curve fit between variables. Blue bands represent the 95% of confidence interval from the fit. Age, race, education, physical activity, smoking behavior, alcohol behavior, BMI, hypertension, diabetes, HbAlc, albumin, blood urea nitrogen, total calcium, cholesterol, creatinine, phosphorus, triglycerides, and uric acid were adjusted.

**Figure 3:**
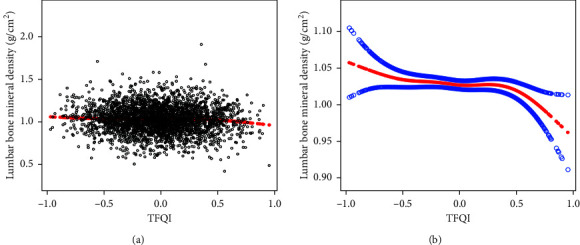
The association between TFQI and lumbar BMD. (a) Each black point represents a sample. (b) Solid red line represents the smooth curve fit between variables. Blue bands represent the 95% of confidence interval from the fit. Age, race, education, physical activity, smoking behavior, alcohol behavior, BMI, hypertension, diabetes, HbAlc, albumin, blood urea nitrogen, total calcium, cholesterol, creatinine, phosphorus, triglycerides, and uric acid were adjusted.

**Figure 4:**
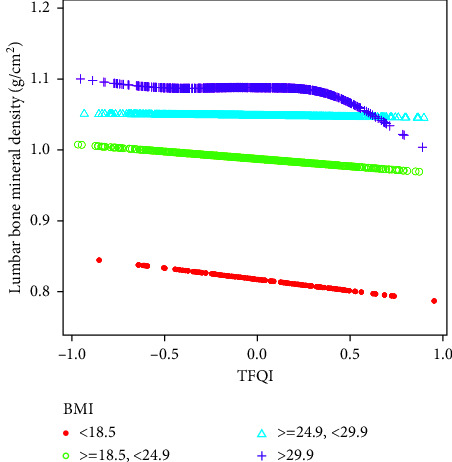
The association between TFQI and lumbar bone mineral density stratified by BMI. Age, race, education, physical activity, smoking behavior, alcohol behavior, BMI, hypertension, diabetes, HbAlc, albumin, blood urea nitrogen, total calcium, cholesterol, creatinine, phosphorus, triglycerides, and uric acid were adjusted.

Smooth curve fitting and generalized additive model were conducted to further analyze the relationship between FT3/FT4, TFQI, and lumbar BMD. Figures 2-3 show that FT3/FT4 and TFQI were negatively associated with lumbar BMD on the whole, and FT3/FT4 and lumbar BMD showed a linear negative correlation. After stratifying the population by BMI, the negative relationship between TFQI and lumbar BMD remained statistically significant at different BMI stratifications (Figure 4), and the relationship in BMI (18.5–24.9 kg/m^2^) group was consistently significant after adjusting for covariates (Table 3).

## 4. Discussion

This study explored the association between BMD and sensitivity to thyroid hormone in healthy men (mean age, 39.48 years; age range, 12–80 years) based on the NHANES 2007–2010 database. This study found that the indices of central and peripheral sensitivity to thyroid hormones were negatively correlated with BMD. However, the relationship between TSHI, TT4RI, and BMD was weak, as shown in Table 2. In subgroup analyses stratified by BMI, TFQI was most strongly associated with lumbar spine BMD in the BMI (18.5–24.9 kg/m^2^) group. In this study, we directly linked resistance to thyroid hormone with bone metabolism, and we hypothesized that osteoporosis can be prevented and treated by modifying thyroid sensitivity.

Thyroid hormones can not only promote the differentiation of osteoblasts and chondrocytes but also directly or indirectly affect the absorption of calcium, phosphorus, and osteogenic substances [33]. Numerous studies have demonstrated that hyperthyroidism, hypothyroidism, TSH suppression therapy for thyroid cancer, and other thyroid diseases may have adverse effects on skeletal development, maintenance, and bone metabolism [34]. According to Svensson et al., there was a strong association between subclinical hyperthyroidism and an increased risk of vertebral fractures in elderly Swedish men [35]. Also, other studies reported that untreated patients with hyperthyroidism had reduced BMD and an increased risk of hip fracture [36, 37]. Conversely, reduced levels of thyroid hormones might lead to decreased stimulation of both osteoblasts and osteoclasts, which affect bone turnover and bone mineralization [38]. Several studies have recently shown a correlation between hypothyroidism and fracture, and patients with a history of hypothyroidism have 2-3 times higher fracture risk [39, 40].

Elevated FT4 and FT3 levels were associated with decreased BMD, and higher FT4 levels might accelerate the loss of the hip bone [41, 42]. In the clinical study of Ale, the levels of FT3 and FT4 in the osteoporosis group and the osteopenia group were higher than those in the normal group [43]. In addition, TSH level is also one of the main pathogenic factors of osteoporosis [44]. Abrahamsen et al. discovered that low TSH levels at baseline may increase the risk of hip fractures [45]. In a study of Korean men, BMD was significantly associated with low serum TSH levels after multivariable adjustment [46]. Nevertheless, in a large cohort of middle-aged men, the result indicated that there was no apparent association between subclinical hypothyroidism or subclinical hyperthyroidism and the BMD at the lumbar spine and femur [47]. As our study showed, there was no significant association between TSH and BMD in men. It may be due to differences in age, gender, race, or location of bone mineral density measurements. Considering the complex interactions in the HPT axis, we proposed to use composite indices instead of individual parameter to reflect the true thyroid status [48].

In thyroid hormone metabolism, T4 is mainly converted to bioactive T3 by deiodinases (DIO), and the FT3/FT4 ratio serves as a proxy of DIO activity and peripheral thyroid hormone sensitivity [49]. According to Nie et al., FT3/FT4 levels are positively correlated with serum osteocalcin levels [50], suggesting the underlying effect of FT3/FT4 on bone remodeling [51]. And relatively higher serum osteocalcin levels have been clinically proved in patients with hyperthyroidism and high turnover osteoporosis [52]. Similarly, the results of this work indicated that FT3/FT4 showed a significant negative association with lumbar spine BMD in males. In terms of central sensitivity to thyroid hormones, both TSHI and TT4RI [53] showed a negative association with BMD; nevertheless, the relationship was weak. TFQI, a novel index that is more stable than TSHI and TT4RI in evaluating thyroid hormone sensitivity [17], was also negatively correlated with the BMD of the lumbar spine, total femur, and femoral neck. After adjusting for multiple confounding factors, the correlation was still significant, suggesting the potential effects of central thyroid hormone resistance on the progression of osteoporosis. Furthermore, as shown in Table 3, a significant association between TFQI and the BMD of the lumbar spine appeared in the male participants with normal BMI (18.5–24.9 kg/m^2^). Therefore, we believed that the dysfunction of peripheral and central response to thyroid hormone might contribute to the disorder of bone metabolism and bone loss, and FT3/FT4 and TFQI were the preferable indicators. As is well known, thyroid hormone action is mediated by thyroid hormone receptor (TR), two major classes of which are known, TR*α* and TR*β*, and TR*α* was considered as the main functional mediator of T3 in the skeleton. This work indicated that increased conversion of FT4 to FT3 exists in osteoporosis patients and elevated FT3 stimulates osteoclastic bone resorption regulated primarily by TR*α*. Moreover, TFQI was also reported to be significantly related to obesity, metabolic syndrome, cardiovascular disease, and diabetes [54, 55], which were also known risk factors for osteoporosis. Hence, the exact mechanisms need to be confirmed in future studies.

In the subgroup analysis, TFQI was negatively correlated with lumbar BMD in different BMI subgroups, and participants in the high BMI group had higher BMD compared with the low BMI group. Furthermore, as reported in Table 3, a significant association between TFQI and the BMD of the lumbar spine appeared in the male participants with normal BMI (18.5–24.9 kg/m^2^). Similarly, a cohort study of 845 individuals reported that patients with high BMI had a higher BMD and protective effect of a high BMI on femoral neck BMD among elderly subjects [56]. Another study by Saarelainen et al. indicated that low BMI was associated with the increased prevalence of osteoporosis and increased fracture risk [57]. Furthermore, Zhang and Pu reported not only a significant positive connection between BMI and BMD but also a BMI saturation value (about 24.3 kg/m^2^) for femoral neck BMD [58]. This might be due to the specific bone-adipose axis that exists between adipose and bone tissue in the body and is connected by various bioactive chemicals to maintain skeletal homeostasis [59].

In our research, we utilized the generalizability of NHANES data, which contained representative noninstitutionalized Americans, which allowed our findings to be presented as generalizability. But we also found limitations in our study. First, we did not collect the antiosteoporotic treatment history of our participants, which affected the objectivity of BMD measurements. In addition, only male data were included in order to eliminate the effects of estrogen. Further studies can investigate the relationship between BMD and sensitivity to thyroid hormone in peri- and postmenopausal women. Last but not least, we could not collect follow-up data in this study to explore the causal relationship between sensitivity to thyroid hormone and osteoporosis, since it was a cross-sectional study.

## 5. Conclusions

Among male representatives of the US population, our study concludes that FT3/FT4 and TFQI are negatively associated with BMD after multiple adjustments. Thus, FT3/FT4 and TFQI are considered to be indicators of the complex relationship between thyroid hormone resistance and bone loss. Moreover, thorough prospective investigations are suggested to confirm these findings.

## Figures and Tables

**Table 1:** Baseline characteristics of the research population based on lumbar spine BMD quartiles.

Characteristics	Quartiles of lumbar spine BMD				*P* value
	Q1 ≤ 0.93	0.93 < Q2 ≤ 1.03	1.03 < Q3 ≤ 1.13	Q4 > 1.13	
Demographics					
Age (years)	33.81 ± 21.11	38.74 ± 18.64	39.98 ± 18.20	44.47 ± 19.05	<0.001

Race/ethnicity (%)					<0.001
Non-Hispanic white	203 (27.92)	174 (23.61)	148 (18.81)	110 (12.85)	
Non-Hispanic black	298 (40.99)	314 (42.61)	363 (46.13)	384 (44.86)	
Mexican American	87 (11.97)	111 (15.06)	157 (19.95)	242 (28.27)	
Other race	139 (19.12)	138 (18.73)	119 (15.12)	120 (14.02)	

Level of education (%)					<0.001
Less than 9th grade	65 (8.94)	87 (11.81)	75 (9.53)	65 (7.59)	
9–11th grade	70 (9.63)	116 (15.74)	119 (15.12)	114 (13.318)	
High school grad	119 (16.37)	140 (19.00)	156 (19.82)	210 (24.53)	
Some college	89 (12.242)	132 (17.91)	161 (20.46)	213 (24.88)	
College graduate or above	67 (9.22)	103 (13.98)	141 (17.92)	167 (19.51)	
Missing	317 (43.60)	159 (21.57)	135 (17.15)	87 (10.16)	

Alcohol consumption (%)					<0.001
Nonsignificant alcohol intake	309 (42.50)	466 (63.23)	535 (67.98)	627 (73.25)	
Significant alcohol intake	418 (57.49)	271 (36.97)	252 (32.02)	229 (26.75)	
Smoking behavior (%)					<0.001
Significant smoking behavior	231 (31.77)	339 (46.00)	341 (43.33)	403 (47.08)	
Never or nonsignificant smoking	496 (68.23)	398 (54.00)	446 (56.67)	453 (52.92)	

Physical activity (%)					0.156
Light	112 (15.41)	138 (18.73)	139 (17.662)	166 (19.39)	
Moderate	145 (19.95)	112 (15.20)	157 (19.95)	159 (18.58)	
High	49 (6.74)	41 (5.56)	53 (6.74)	53 (6.19)	
Not recorded	421 (57.91)	446 (60.52)	438 (55.65)	478 (55.84)	

BMI (kg/m^2^)	24.29 ± 5.14	26.49 ± 5.16	27.60 ± 5.06	28.86 ± 4.92	<0.001

Hypertension (%)	103 (14.17)	137 (18.59)	188 (23.89)	273 (31.89)	<0.001

Diabetes (%)	42 (5.78)	53 (7.19)	57 (7.24)	86 (10.05)	0.035

Laboratory indices					
HbA1c (%)	5.49 ± 0.94	5.60 ± 1.07	5.60 ± 0.98	5.69 ± 1.01	<0.001
Albumin (g/dL)	4.41 ± 0.31	4.41 ± 0.30	4.39 ± 0.31	4.35 ± 0.3	0.002
Blood urea nitrogen (mg/dL)	11.98 ± 4.12	12.57 ± 4.42	12.93 ± 4.59	13.48 ± 5.34	<0.001
Total calcium (mg/dL)	9.52 ± 0.36	9.49 ± 0.36	9.50 ± 0.36	9.41 ± 0.36	<0.001
Total cholesterol (mg/dL)	191.32 ± 45.17	192.15 ± 40.66	189.81 ± 40.15	190.53 ± 38.90	0.243
Triglycerides (mg/dL)	149.03 ± 145.31	163.37 ± 166.56	160.98 ± 125.17	168.62 ± 153.68	<0.001
Creatinine (mg/dL)	0.82 ± 0.22	0.94 ± 0.36	0.95 ± 0.20	1.02 ± 0.41	<0.001
Phosphorus (mg/dL)	4.20 ± 0.83	3.78 ± 0.64	3.74 ± 0.61	3.74 ± 0.59	<0.001
Uric acid (mg/dL)	5.61 ± 1.21	5.93 ± 1.25	6.06 ± 1.26	6.19 ± 1.30	<0.001

Thyroid parameters					
FT3 (pmol/L)	5.51 ± 0.81	5.26 ± 0.64	5.16 ± 0.62	5.04 ± 0.61	<0.001
FT4 (pmol/L)	10.25 ± 1.72	10.23 ± 1.87	10.04 ± 1.76	10.03 ± 1.86	0.006
TSH (mIU/L)	1.96 ± 3.19	1.77 ± 1.39	1.76 ± 1.27	1.93 ± 2.42	0.202
FT3/FT4	0.55 ± 0.12	0.53 ± 0.10	0.53 ± 0.09	0.52 ± 0.10	<0.001
TSHI	1.81 ± 0.66	1.75 ± 0.63	1.72 ± 0.65	1.76 ± 0.67	0.124
TT4RI	19.18 ± 16.67	17.80 ± 13.72	17.36 ± 11.42	18.35 ± 13.98	0.131
TFQI	0.01 ± 0.33	−0.02 ± 0.32	−0.04 ± 0.33	−0.05 ± 0.33	<0.001

Mean ± SD for continuous variables: the *P* value was calculated by the weighted linear regression model. (%) for categorical variables: the *P* value was calculated by the weighted chi-square test. BMD, bone mineral density; BMI, body mass index; HbA1c, glycosylated hemoglobin A1c; FT3, serum free triiodothyronine; FT4, free thyroxine; TSH, thyroid-stimulating hormone; FT3/FT4, free triiodothyronine/free thyroxine; TSHI, thyroid-stimulating hormone index; TT4RI, thyrotroph thyroxine resistance index; TFQI, thyroid feedback quantile-based index.

**Table 2:** The correlation between sensitivity to thyroid hormone indices and BMD in all parts of the body.

*β* (95% CI), *P* value	FT3/FT4	TFQI	TSHI	TT4RI
Lumbar spine BMD				
Model 1	−0.205 (−0.257, −0.154)^*∗∗∗*^	−0.027 (−0.044, −0.010)^*∗∗*^	−0.009 (−0.017, 0.000)	−0.000 (−0.001, 0.000)
Model 2	−0.102 (−0.155, −0.050)^*∗∗∗*^	−0.022 (−0.038, −0.005)^*∗∗*^	−0.009 (−0.017, −0.001)^*∗*^	−0.000 (−0.001, −0.000)^*∗*^
Model 3	−0.035 (−0.084, −0.013)^*∗*^	−0.018 (−0.033, −0.003)^*∗*^	−0.008 (−0.015, −0.000)^*∗*^	−0.000 (−0.001, −0.000)^*∗*^

Total femur BMD				
Model 1	0.039 (−0.012, 0.090)	−0.040 (−0.056, −0.023)^*∗∗∗*^	−0.015 (−0.023, −0.007)^*∗∗∗*^	−0.001 (−0.001, −0.000)^*∗*^
Model 2	−0.037 (−0.089, 0.016)	−0.020 (−0.036, −0.003)^*∗*^	−0.005 (−0.013, −0.003)^*∗*^	−0.000 (−0.001, 0.000)
Model 3	−0.011 (−0.059, 0.036)	−0.020 (−0.035, −0.006)^*∗∗*^	−0.008 (−0.015, −0.001)^*∗*^	−0.000 (−0.001, −0.000)^*∗∗*^

Femoral neck BMD				
Model 1	0.065 (0.021, 0.109)^*∗∗*^	−0.028 (−0.043, −0.014)^*∗∗∗*^	−0.011 (−0.018, −0.004)^*∗∗*^	−0.001 (−0.001, −0.000)^*∗*^
Model 2	−0.004 (−0.049, −0.001)	−0.013 (−0.027, −0.001)^*∗∗*^	−0.003 (−0.010, −0.004)^*∗*^	−0.000 (−0.001, 0.000)
Model 3	0.007 (−0.036, 0.050)	−0.018 (−0.031, −0.005)^*∗∗*^	−0.008 (−0.014, −0.001)^*∗*^	−0.001 (−0.001, −0.000)^*∗∗*^

Logistic regression models: Model 1, no covariates were adjusted. Model 2 was adjusted for demographic factors, including age, race, and education. Model 3 was adjusted for age, race, education, physical activity, smoking behavior, alcohol behavior, BMI, hypertension, diabetes, HbA1c, albumin, blood urea nitrogen, total calcium, cholesterol, creatinine, phosphorus, triglycerides, and uric acid. *P*^*∗*^ < 0.05; *P*^*∗∗*^ < 0.01; *P*^*∗∗∗*^ < 0.001.

**Table 3:** Association between sensitivity to thyroid hormone indices and lumbar spine BMD.

	Model 1	Model 2	Model 3
	*β* (95% CI)	*β* (95% CI)	*β* (95% CI)
TFQI (quartile)			
Q1	Reference	Reference	Reference
Q2	−0.016 (−0.032, −0.001)^*∗*^	−0.006 (−0.021, −0.009)^*∗*^	−0.005 (−0.019, −0.008)^*∗*^
Q3	−0.015 (−0.031, −0.000)^*∗*^	−0.007 (−0.022, 0.008)	−0.010 (−0.024, −0.003)^*∗*^
Q4	−0.022 (−0.038, −0.007)^*∗∗*^	−0.015 (−0.030, −0.000)^*∗*^	−0.012 (−0.025, −0.002)^*∗*^

*P* for tend	0.008	0.005	0.006

Subgroup analysis stratified by BMI (kg/m^2^)			
BMI < 18.5	−0.034 (−0.109, 0.041)	−0.024 (−0.089, 0.041)	0.001 (−0.063, 0.066)
BMI 18.5–24.9	−0.045 (−0.072, −0.019)^*∗∗∗*^	−0.026 (−0.046, −0.006)^*∗*^	−0.025 (−0.044, −0.005)^*∗*^
BMI 25–29.9	−0.009 (−0.035, 0.017)	0.007 (−0.019, 0.033)	−0.005 (−0.030, 0.021)
BMI ≥ 30	−0.026 (−0.057, −0.005)^*∗*^	−0.020 (−0.051, 0.010)	−0.022 (−0.052, 0.008)

Logistic regression models: Model 1, no covariates were adjusted. Model 2 was adjusted for demographic factors, including age, race, and education. Model 3 was adjusted for age, race, education, physical activity, smoking behavior, alcohol behavior, BMI, hypertension, diabetes, HbA1c, albumin, blood urea nitrogen, total calcium, cholesterol, creatinine, phosphorus, triglycerides, and uric acid. *P*^*∗*^ < 0.05; *P*^*∗∗*^ < 0.01; *P*^*∗∗∗*^ < 0.001.

## Data Availability

The survey data are publicly available on the Internet for data users and researchers throughout the world https://www.cdc.gov/nchs/nhanes/.

## References

[1] Kanis J. A., Cooper C., Rizzoli R., Reginster J. Y. (2019). European guidance for the diagnosis and management of osteoporosis in postmenopausal women. *Osteoporosis International*.

[2] Zhang X., Chen K., Chen X. (2020). Integrative analysis of genomics and transcriptome data to identify regulation networks in female osteoporosis. *Front Genet*.

[3] Wright N. C., Looker A. C., Saag K. G. (2014). The recent prevalence of osteoporosis and low bone mass in the United States based on bone mineral density at the femoral neck or lumbar spine. *Journal of Bone and Mineral Research*.

[4] Tanha K., Fahimfar N., Nematollahi S. (2021). Annual incidence of osteoporotic hip fractures in Iran: a systematic review and meta-analysis. *BMC Geriatr*.

[5] Williams S. A., Daigle S. G., Weiss R., Wang Y., Arora T., Curtis J. R. (2021). Economic burden of osteoporosis-related fractures in the US medicare population. *Ann. Pharmacother.*.

[6] Lane N. E. (2006). Epidemiology, etiology, and diagnosis of osteoporosis. *American Journal of Obstetrics and Gynecology*.

[7] Xu F., Li W., Yang X., Na L., Chen L., Liu G. (2020). The roles of epigenetics regulation in bone metabolism and osteoporosis. *Frontiers in Cell and Developmental Biology*.

[8] Williams G. R., Bassett J. H. D. (2018). Thyroid diseases and bone health. *Journal of Endocrinological Investigation*.

[9] Viapiana O., Fracassi E., Troplini S. (2013). Sclerostin and DKK1 in primary hyperparathyroidism. *Calcified Tissue International*.

[10] Bassett J. H. D., Williams G. R. (2016). Role of thyroid hormones in skeletal development and bone maintenance. *Endocrine Reviews*.

[11] Mosekilde L., Eriksen E. F., Charles P. (1990). Effects of thyroid hormones on bone and mineral metabolism. *Endocrinology and Metabolism Clinics of North America*.

[12] Yang S., Lai S., Wang Z., Liu A., Wang W., Guan H. (2021). Thyroid Feedback quantile-based Index correlates strongly to renal function in euthyroid individuals. *Annals of Medicine*.

[13] van der Spek A. H., Fliers E., Boelen A. (2017). The classic pathways of thyroid hormone metabolism. *Molecular and Cellular Endocrinology*.

[14] Jostel A., Ryder W. D. J., Shalet S. M. (2009). The use of thyroid function tests in the diagnosis of hypopituitarism: definition and evaluation of the TSH Index. *Clinical Endocrinology*.

[15] Susiarjo M., Xin F., Bansal A. (2015). Bisphenol a exposure disrupts metabolic health across multiple generations in the mouse. *Endocrinology*.

[16] Hoermann R., Midgley J. E. M., Larisch R., Dietrich J. W. (2018). The role of functional thyroid capacity in pituitary thyroid feedback regulation. *European Journal of Clinical Investigation*.

[17] Laclaustra M., Moreno-Franco B., Lou-Bonafonte J. M. (2019). Impaired sensitivity to thyroid hormones is associated with diabetes and metabolic syndrome. *Diabetes Care*.

[18] Adams J. E. (2013). Advances in bone imaging for osteoporosis. *Nature Reviews Endocrinology*.

[19] Liu H., Paige N. M., Goldzweig C. L. (2008). Screening for osteoporosis in men: a systematic review for an American college of physicians guideline. *Annals of Internal Medicine*.

[20] Hollowell J. G., Staehling N. W., Flanders W. D. (2002). Serum TSH, T(4), and thyroid antibodies in the United States population (1988 to 1994): national health and nutrition examination survey (NHANES III). *Journal of Clinical Endocrinology & Metabolism*.

[21] Sun Y., Xia P. F., Korevaar T. I. M. (2021). Relationship between blood trihalomethane concentrations and serum thyroid function measures in U.S. adults. *Environmental Science and Technology*.

[22] Liu N., Ma F., Feng Y., Ma X. (2021). The association between the dietary inflammatory index and thyroid function in U.S. Adult males. *Nutrients*.

[23] Nie X., Ma X., Xu Y., Shen Y., Wang Y., Bao Y. (2020). Increased serum adipocyte fatty acid-binding protein levels are associated with decreased sensitivity to thyroid hormones in the euthyroid population. *Thyroid*.

[24] Yagi H., Pohlenz J., Hayashi Y., Sakurai A., Refetoff S. (1997). Resistance to thyroid hormone caused by two mutant thyroid hormone receptors *β*, R243Q and R243W, with marked impairment of function that cannot Be explained by altered*in Vitro*3, 5, 3′-triiodothyroinine binding Affinity. *Journal of Clinical Endocrinology & Metabolism*.

[25] Shen Q., Xu Q., Li G. (2021). Joint effect of 25-hydroxyvitamin D and secondhand smoke exposure on hypertension in non-smoking women of childbearing age: NHANES 2007–2014. *Environmental Health*.

[26] Agrawal P., Mercer A., Hassanali J., Carmack C., Doss D., Murillo R. (2018). Gender differences in the association between alcohol use and sedentary behavior among adults. *American Journal of Health Promotion*.

[27] Cao C., Friedenreich C. M., Yang L. (2022). Association of daily sitting time and leisure-time physical activity with survival among US cancer survivors. *JAMA Oncology*.

[28] Piercy K. L., Troiano R. P., Ballard R. M. (2018). The physical activity Guidelines for Americans. *JAMA*.

[29] Ma M., Feng Z., Liu X., Jia G., Geng B., Xia Y. (2021). The saturation effect of body mass index on bone mineral density for people over 50 years old: a cross-sectional study of the US population. *Frontiers in Nutrition*.

[30] Jung M. H., Youn H. J., Ihm S. H., Jung H. O., Hong K. S. (2018). Heart rate and bone mineral density in older women with hypertension: results from the Korea national health and nutritional examination survey. *Journal of the American Geriatrics Society*.

[31] Yuan J., Jia P., Zhou J. B. (2022). Comparison of bone mineral density in US adults with diabetes, prediabetes and normoglycemia from 2005 to 2018. *Frontiers in Endocrinology*.

[32] Stookey J. D. (2019). Analysis of 2009–2012 nutrition health and examination survey (NHANES) data to estimate the median water intake associated with meeting hydration criteria for individuals aged 12–80 in the US population. *Nutrients*.

[33] Delitala A. P., Scuteri A., Doria C. (2020). Thyroid hormone diseases and osteoporosis. *Journal of Clinical Medicine*.

[34] Biondi B., Cooper D. S. (2019). Thyroid hormone suppression therapy. *Endocrinology and Metabolism Clinics of North America*.

[35] Svensson J., Ohlsson C., Karlsson M. K., Lorentzon M., Lewerin C., Mellstrom D. (2021). Subclinical hyperthyroidism is associated with increased risk of vertebral fractures in older men. *Osteoporosis International*.

[36] Liu H., Ma Q., Han X., Huang W. (2020). Bone mineral density and its correlation with serum 25-hydroxyvitamin D levels in patients with hyperthyroidism. *Journal of International Medical Research*.

[37] Vestergaard P., Mosekilde L. (2003). Hyperthyroidism, bone mineral, and fracture risk--a meta-analysis. *Thyroid*.

[38] Apostu D., Lucaciu O., Oltean-Dan D. (2020). The influence of thyroid pathology on osteoporosis and fracture risk: a review. *Diagnostics*.

[39] Rosario P. W., Carvalho M., Calsolari M. R. (2016). Symptoms of thyrotoxicosis, bone metabolism and occult atrial fibrillation in older women with mild endogenous subclinical hyperthyroidism. *Clinical Endocrinology*.

[40] Garmendia Madariaga A., Santos Palacios S., Guillen-Grima F., Galofre J. C. (2014). The incidence and prevalence of thyroid dysfunction in Europe: a meta-analysis. *Journal of Clinical Endocrinology & Metabolism*.

[41] Mogharbel B. F., Abdelwahid E., Irioda A. C. (2017). Bone marrow-derived stem cell populations are differentially regulated by thyroid or/and ovarian hormone loss. *International Journal of Molecular Sciences*.

[42] Murphy E., Gluer C. C., Reid D. M. (2010). Thyroid function within the upper normal range is associated with reduced bone mineral density and an increased risk of nonvertebral fractures in healthy euthyroid postmenopausal women. *Journal of Clinical Endocrinology & Metabolism*.

[43] Ale A. O., Ogbera A. O., Ebili H. O., Adeyemo O. L., Afe T. O. (2018). Prevalence, predictive factors, and characteristics of osteoporosis in hyperthyroid patients. *International Journal of Endocrinology*.

[44] Sun L., Davies T. F., Blair H. C., Abe E., Zaidi M. (2006). TSH and bone loss. *Annals of the New York Academy of Sciences*.

[45] Abrahamsen B., Jorgensen H. L., Laulund A. S., Nybo M., Brix T. H., Hegedus L. (2014). Low serum thyrotropin level and duration of suppression as a predictor of major osteoporotic fractures-the openthyro register cohort. *Journal of Bone and Mineral Research*.

[46] Kim B. J., Lee S. H., Bae S. J. (2010). Original article: the association between serum thyrotropin (TSH) levels and bone mineral density in healthy euthyroid men. *Clinical Endocrinology*.

[47] Lee K., Lim S., Park H. (2020). Subclinical thyroid dysfunction, bone mineral density, and osteoporosis in a middle-aged Korean population. *Osteoporosis International*.

[48] Lai S., Li J., Wang Z., Wang W., Guan H. (2021). Sensitivity to thyroid hormone indices are closely associated with NAFLD. *Frontiers in Endocrinology*.

[49] Haddow J. E., Craig W. Y., Neveux L. M. (2014). Implications of high free thyroxine (FT4) concentrations in euthyroid pregnancies: the FaSTER trial. *Journal of Clinical Endocrinology & Metabolism*.

[50] Nie X., Xu Y., Shen Y., Wang Y., Ma X., Bao Y. (2021). Suppressing effect of free triiodothyronine on the negative association between body mass index and serum osteocalcin levels in euthyroid population. *International Journal of Endocrinology*.

[51] Zhou J., Liu B., Qin M. Z., Liu J. P. (2020). Fall prevention and anti-osteoporosis in osteopenia patients of 80 Years of age and older: a randomized controlled study. *Orthopaedic Surgery*.

[52] Li G., Jiang X., Liu L., Liu X., Liu H., Zhang Z. (2019). Effect of estradiol on high glucoseinduced osteoblast injury. *Molecular Medicine Reports*.

[53] Dietrich J. W., Landgrafe-Mende G., Wiora E. (2016). Calculated parameters of thyroid homeostasis: emerging tools for differential diagnosis and clinical research. *Frontiers in Endocrinology*.

[54] Sun Y., Teng D., Zhao L. (2022). Impaired sensitivity to thyroid hormones is associated with hyperuricemia, obesity, and cardiovascular disease risk in subjects with subclinical hypothyroidism. *Thyroid*.

[55] Mehran L., Delbari N., Amouzegar A., Hasheminia M., Tohidi M., Azizi F. (2022). Reduced sensitivity to thyroid hormone is associated with diabetes and hypertension. *Journal of Clinical Endocrinology & Metabolism*.

[56] Barrera G., Bunout D., Gattas V., de la Maza M. P., Leiva L., Hirsch S. (2004). A high body mass index protects against femoral neck osteoporosis in healthy elderly subjects. *Nutrition*.

[57] Saarelainen J., Kiviniemi V., Kroger H. (2012). Body mass index and bone loss among postmenopausal women: the 10-year follow-up of the OSTPRE cohort. *Journal of Bone and Mineral Metabolism*.

[58] Zhang Y., Pu J. (2022). The saturation effect of obesity on bone mineral density for older people: the NHANES 2017–2020. *Frontiers in Endocrinology*.

[59] Gomez-Ambrosi J., Rodriguez A., Catalan V., Fruhbeck G. (2008). The bone-adipose axis in obesity and weight loss. *Obesity Surgery*.

